# Factors influencing return to work 3 months after percutaneous coronary intervention in young and middle-aged patients with coronary heart disease: A single-center, cross-sectional study

**DOI:** 10.1371/journal.pone.0284100

**Published:** 2023-04-19

**Authors:** Yaoyao Hu, Taihua Zhou, Xiaojing Li, Xiaoxiao Chen, Xiaoyan Wang, Jiahui Xu, Danfeng Gu

**Affiliations:** 1 Department of Cardiology, The Affiliated Hospital of Jiangnan University, Wuxi, Jiangsu, China; 2 Nursing Department, The Affiliated Hospital of Jiangnan University, Wuxi, Jiangsu, China; Azienda Ospedaliero Universitaria Careggi, ITALY

## Abstract

**Background:**

Today, patients with coronary heart disease (CHD) are becoming younger and younger, and after percutaneous coronary intervention (PCI), most patients want to resume their occupations. The return to work of patients with CHD post PCI in China, however, has received little research attention. So, the goal of this study was to investigate the variables impacting the return to work following PCI in young and middle-aged patients with CHD in Wuxi and to offer a reference basis for the development of targeted interventions.

**Methods:**

This study was executed at the Affiliated Hospital of Jiangnan University. We selected 280 young and middle-aged patients who underwent PCI for CHD as the study subjects and gathered general data about them while they were hospitalized. At 3 months after PCI, we surveyed the subjects with the return to work self-efficacy questionnaire, the Chinese version of the brief fatigue inventory, and the social support rating scale, and obtained information about their return to work. The factors affecting patients’ returning to work were analyzed using binary logistic regression.

**Results:**

The final 255 cases were included in the study, of which 155 (60.8%) were successfully returned to work. Binary logistic regression showed that women (OR = 0.379, 95%CI:0.169,0.851), ejection fraction ≥50% (OR = 2.053, 95%CI:1.085,3.885), the brain-based job types (OR = 2.902, 95%CI:1.361,6.190), the kind of employment requiring both mental and physical capacity (OR = 2.867, 95%CI:1.224,6.715), moderate fatigue (OR = 6.023, 95%:1.596,22.7251), mild fatigue (OR = 4.035, 95%:1.104,14.751), return to work efficacy (OR = 1.839, 95%:1.140,3.144), and social support (OR = 1.060, 95%:1.003,1.121) were independent influences on patients’ return to work at 3 months after PCI (All *P*<0.05).

**Conclusion:**

In order to help patient return to work as soon as possible, healthcare professionals should focus on those who are female, have worked mainly in physical activities, have low return-to-work self-efficacy, have severe fatigue, have low social support, and have poor ejection fraction.

## Introduction

Coronary heart disease (CHD) is a myocardial ischemic and hypoxic disease caused by narrowing or blockage of coronary arteries, and is featured by high morbidity, high mortality and youthfulness [1]. According to the American Heart Association, the number of deaths from cardiovascular disease worldwide is expected to exceed 23.6 million by 2030, and the economic burden of cardiovascular disease will exceed $1 trillion [2]. The prevalence of CHD among young and middle-aged people, as well as the morbidity and mortality rates, and also the economic cost and other issues that follow, are all increasing over the years, according to domestic and international studies [3, 4].

Percutaneous coronary intervention (PCI) is a percutaneous puncture technique to deliver a balloon catheter or other related device to relieve coronary artery stenosis or obstruction and reestablish coronary blood flow, used for the treatment of clinical high-risk patients (acute coronary syndromes, medical comorbidities, etc.) and a variety of complex coronary lesions, and is widely utilized in clinical practice [5]. Depending on the timing of the procedure and the degree of urgency of the onset of CHD, PCI can be divided into emergency PCI and elective PCI [6]. For patients with stable coronary heart disease, PCI can be electively performed after a detailed preoperative evaluation and preparation. Emergency PCI is suitable for patients with acute coronary syndromes within the time window of the procedure. Relevant data revealed that the total number of PCI in mainland China in 2018 was 915,256 cases, and the average number of cases per million population in China in 2018 was 651 [1]. Regardless of the PCI procedure, it can improve long-term survival, quality of life, and lessen clinical symptoms related to myocardial ischemia in individuals with CHD. Naturally, this also involves people with CHD who are young or middle-aged who have increased survival after PCI. So, we need to be concerned about this group of people.

The young and middle-aged population is special because they have important social, family and life responsibilities, which is why a growing number of young and middle-aged patients with CHD aspire to return to work and regain some social role after PCI [7]. Returning to work means that patients return to their previous jobs or accept new paid jobs, which is important for improving patients’ quality of life and achieving self-worth, and is also one of the important indicators of disease recovery [8–10]. A study of 3291 patients with CHD in 24 European countries found that 76% of patients could return to work within 6 months to 3 years after discharge [9]. A prospective cohort study carried out by Norwegian scholars found that approximately 89% of patients returned to work 3 years after PCI [11]. The results of a questionnaire survey conducted by Wang on 428 patients 3 years after PCI revealed that 5.7% of patients were unable to work or had serious health problems at work, and 58.8% of patients had a significant decrease in work level and needed to reduce their work hours [12]. An investigation of 1165 patients treated with PCI in 21 provinces in China found that 55.9% of patients returned to work within 1 year after an acute myocardial infarction [13]. It can be seen from the above that, compared with the data of those returning to work abroad, the rate of return to work of young and middle-aged patients with CHD in China is relatively low. With the trend of younger onset of CHD and the improvement of survival rate in China, young and middle-aged patients with CHD may become a population that needs to be paid attention to when returning to work. Thus, it is important to understand the factors affecting patients’ return to work following PCI and to take action that may have benefits for that return to work.

The process of returning to the workforce is complex and multidimensional, previous studies have validated the influencing variables or predictors of returning to work after PCI. The cardio-related factors (left ventricular ejection fraction, exercise capacity, treatment/procedural factors, rhythm stability, existing comorbidities, etc.), psychosocial factors (work stress, anxiety, depression, dual work and life stress, etc.), and work-related factors (work intensity, work environment, etc.) that are related to patients with CHD returning to work have all been reviewed in a paper [14]. According to research by Warraich, readmission in the first year following myocardial infarction, baseline smoking, hypertension, and post-discharge bleeding were all linked to negative changes in employment [15]. In addition, Stendardo’s study pointed out that the strongest predictors of return to work within 1 year after discharge from acute myocardial infarction were more related to social, psychological, and occupational factors such as education, employment status, and depression than clinical parameters [16]. According to findings by Worcester, variables related to return to work include the rate with which people participate in cardiac rehabilitation, the length of hospital stays, previous angina, performing manual labor or physically demanding jobs, and job satisfaction [17].

Only a small number of research in China have investigated the variables that affect patients with CHD going back to work. A survey showed that type of disease, ejection fraction, gender, nature of work, fatigue, return-to-work self-efficacy, and family function were related to patients’ return-to-work at 3 months after discharge [18]. Another investigation additionally showed that interventional therapy, intraoperative and postoperative complications, cardiac function, fear of disease progression, and social support level were the factors that affected patients with early-onset (<45 years old) coronary heart disease’s action to return to work after interventional therapy [19]. Since there are significantly different in the laws, cultural norms, policy supports of different nations with regard to returning to work, as well as currently no standardized guidelines or policies for reintegrating CHD patients into the profession, more and stronger evidence is still required to argue for and look into the variables affecting patients’ getting back to work.

However, few research papers have been published in China on the factors influencing return to work after PCI in young and middle-aged patients. So, this study aimed to explore the factors influencing the return to work after PCI in young and middle-aged patients with CHD, and to offer a reference basis for constructing a return to work intervention scheme in the context of our culture to facilitate their early return to society and family.

## Materials and methods

### Study design and settings

From December 2020 to December 2021, this study was conducted at the Department of Cardiovascular Medicine at the Jiangnan University Affiliated Hospital. Throughout the patients’ hospital stays, we gathered general data for this study on them. Once a month for a total of three months after they were discharged from the hospital, we visited them at their homes, over the phone, or on WeChat to reduce sample loss. At the end of month 3, we collected data on whether patients returned to work and on fatigue, social support, and return-to-work self-efficacy. The ethics committee of the Jiangnan University Affiliated Hospital approved the research (LS2020047), which was performed out in conformity with the Declaration of Helsinki. According to the guidelines for "Strengthening the Reporting of Observational Studies in Epidemiology (STROBE)", the study was designed.

The Department of Cardiovascular Medicine of the Affiliated Hospital of Jiangnan University is the national chest pain center of China and the key clinical specialty unit of Jiangsu Province. The cardiovascular department has 3 cardiovascular wards and 1 coronary care unit, 2 DSA catheterization rooms and 120 beds. On average, the department performs 300 emergency procedures and 80,000 outpatients each year.

### Participants

The samples for this study were selected from the Department of Cardiovascular Medicine, Affiliated Hospital of Jiangnan University, Wuxi, China. The following requirements had to be met in order for a patient to be included in our study: (1) age between 18 and 59 years; (2) initial diagnosis of CHD and PCI treatment; (3) agreement to participate in the study and signature on the paper version of the informed consent form; (4) had a job with a relatively stable salary prior to PCI (include the employed and the self-paid); and (5) ability to communicate and interact normally. The criteria would be used to determine who was excluded from the study: (1) participants with a history of anxiety, depression, mental illness, or cognitive impairment; (2) participants with malignant tumors, advanced liver and kidney diseases, and other serious diseases that damaged survival; (3) participants who developed additional conditions that affected survival during the follow-up period.

Multifactorial regression analyses have shown that the sample size is at least 5–10 times the number of independent variables [20]. The required sample size for this study, which contained 22 independent variables and required 110–220 cases to account for a 20% probability of missing samples, was actually 138–275 cases. After allowing for additional unpredictable survey-process variables, 280 cases were chosen as the final sample size for this study.

**Fig 1:**
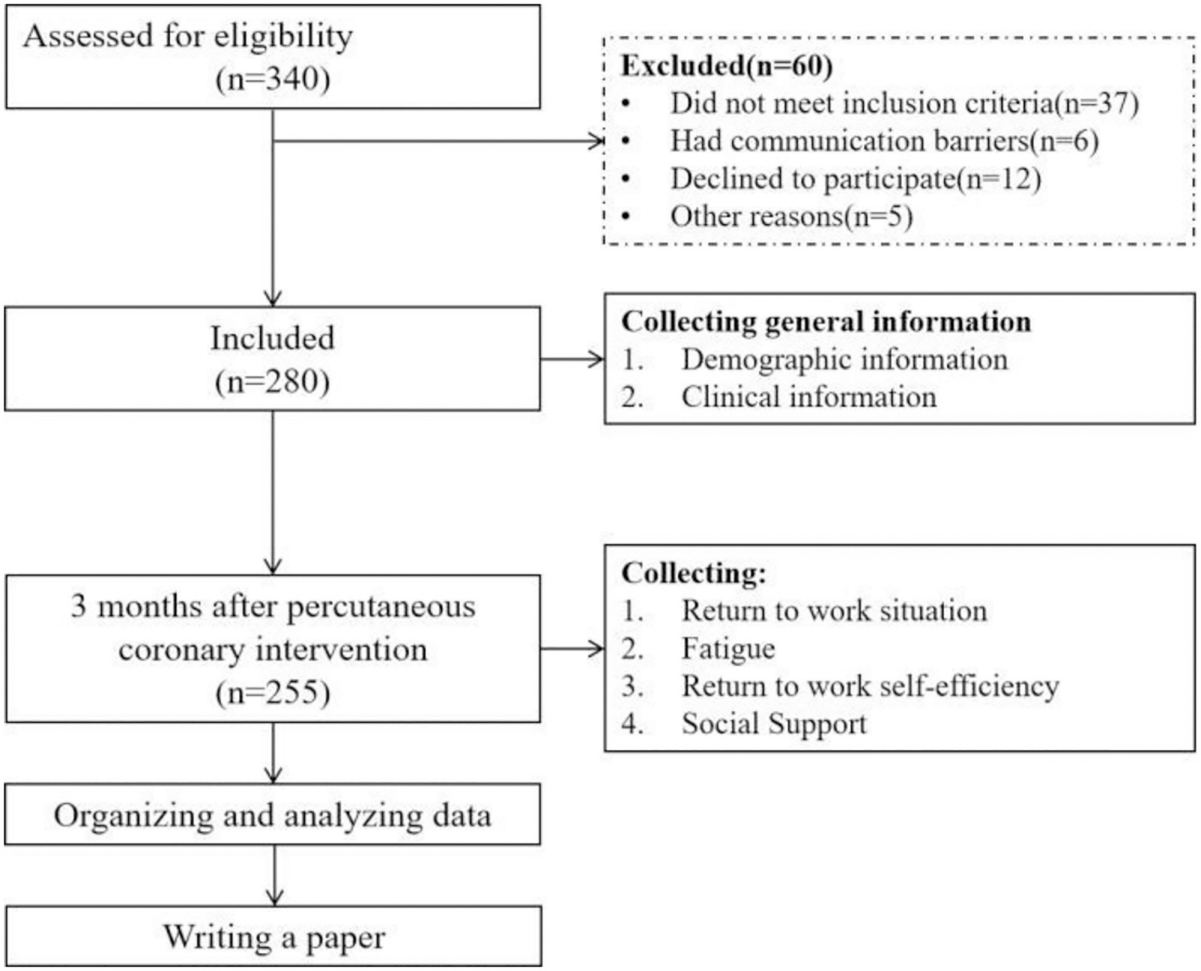
Enrollment and following flowcharts for the whole research project.

In order to obtain participants, we posted advertisements about the study on the WeChat public website, the outpatient gate, and the inpatient cardiology ward’s bulletin board. Due to COVID-19, the full recruitment process took place over the course of about 5 months, from December 2020 to May 2021. After being informed of the purposes and methods of the survey, all voluntary patients signed a paper version of the informed consent form. A total of 340 study participants were recruited, however, 37 did not satisfy the criteria for inclusion, 6 experienced communication issues, 12 declined to participate, and 5 had other excuses, leaving 280 who did. At 3 months after PCI, 9 cases had incorrect cell phone numbers, 10 cases rejected further participation, 1 case developed leukemia, and 4 cases had no answer for 3 consecutive calls, which resulted in 255 subjects being successfully tracked (Fig 1).

### Data collection and instruments

Returning to work is a complex process that is impacted by a wide range of socioeconomic, psychological, physical, and occupational issues. Many studies have been conducted in the field of CHD or other chronic diseases to confirm the influences or predictors of returning to work [14–19, 21–23]. These factors include: age, gender, medical insurance, income, smoking, education, comorbidities, return to work self-efficacy, ejection fraction, type of job, social support, marital status, fatigue, disease type, treatment method, number of diseased vessels, blood lipids, work hours, etc. Combined with the cultural background of our country, the discussion among the members of the research group, and the research purposes, we decided to gather information from socio-demographic (age, gender, education level, marital status, monthly income, medical insurance, smoking history, drinking history, social support, body mass index), disease-related data (diagnosis, type of PCI, length of hospital stay, blood lipid, number of lesion vessels, ejection fraction, heart function, medication, recurrence), physiological and psychological (fatigue, return to work self-efficacy), and occupation (working hours, type of work), to explore the influencing factors of returning to work in young and middle-aged patients with CHD.

Data were collected by our study team, who received uniform training prior to data collection. Our study team included one chief cardiovascular physician, two graduate nursing students, two nurse practitioners in charge, one associate nurse practitioner, and one chief nurse practitioner. The researchers explained the study’s goals, methodology, significance, etc. to the participants before the study began. The researchers only carried out the study well with participants’ written consent. Further, it was assured that all participant data and information would only be used for this study and wouldn’t be shared with anyone else.

The researchers collected demographic data from patients in the inpatient cardiology ward during the post-PCI stabilization phase and clinical data from BayNexus Medical Information System or patients’ medical records. In accordance with the findings of Biering et al., 84.5% (528/625) of patients with ischemic heart disease returned to work at 3 months after PCI [24]. Thus, this study selected to gather data in the cardiology clinic 3 months after PCI (i.e., patient review) on patients’ return to work, fatigue, return to work self-efficacy, and social support. For the purposes of this study, return to work was defined as a patient successfully returning to their previous job or accepting a new paid offer at 3 months after PCI.

A general information questionnaire, designed by the researchers themselves, was used to obtain general information about the patients. It mainly included demographic and sociological information (age, spouse, gender, education level, monthly income, etc.) and clinical information (diagnosis, medications, clinical metabolic indicators, ejection fraction, etc.). Our investigators use the BayNexus Medical Information Platform or medical records to view and capture clinical information on subjects.

The Chinese Version of the Brief Fatigue Inventory (BFI-C) was used to evaluate the fatigue level of patients, which was developed by Mendoza [25]. The instrument has 9 entries, the first 3 of which assess the current level of fatigue, the average and worst levels of fatigue over the past 24h. The 4th-9th items assess the impact of fatigue on different aspects of life. The inventory was evaluated using the 0–10 line method, with 0 indicating none and 10 indicating the most severe, and the final score of the inventory was the mean score of the 10 entries. According to the final score, there are three categories of fatigue: mild fatigue (scores of 1–3), moderate fatigue (scores of 4–6), and severe fatigue (scores of 7–10). For this scale, the Cronbach’s α coefficient was 0.94 [26], when in this study, that was 0.91.

The Return to Work Self-efficacy Questionnaire (RTW-SE) was administered to measure patients’ return to work self-efficacy [27]. The 11-item questionnaire uses a Likert 6-point scale (1 to 6) and asks study participants to rate their statements about their job assuming they return to work tomorrow. The final score on the scale was the mean of the 11 items, with higher scores denoting higher levels of return-to-work self-efficacy. For the Chinese translation of RTW-SE, the Cronbach’s α coefficient was 0.93 [28]. The Cronbach’s α coefficient for RTW-SE in this study was 0.89.

The Social Support Rating Scale (SSRS) was developed by Xiao [29], a domestic scholar, and is widely used in China to measure patients’ social support. The SSRS has 10 items and the total score ranges from 12 to 66, with higher scores indicating better social support. Zhang used this scale for patients with CHD, and the Cronbach’s α coefficient of this scale was 0.84 [30], which was 0.82 in this study.

### Statistical analysis

A researcher who upheld tight confidentiality of the assigned data performed statistical analysis using the SPSS 25.0 software package. Information about counts was given as frequency and percentage. The mean and standard deviation were used to describe continuous measures that matched a normal distribution, and median (P25, P75) was used to describe those that did not. Using independent samples t-tests or Mann-Whitney U-tests, continuous variables were compared between groups of categorical variables. For comparing count information data between groups, the chi-square test, Fisher exact test, and Wilcoxon rank sum test were utilized. The multivariable logistic regression model was used to examine the independent influencing factors of returning to work. A *P* value of 0.05 or lower signifies statistical significance throughout the analysis.

## Results

Initially, 280 young and middle-aged patients with coronary heart disease who met the inclusion and exclusion criteria were selected for the study. During the follow-up process 3 months after PCI, we successfully followed up 255 (91.1%) of these patients, and of the 25 who were not followed up, 10 refused to continue to participate in this study, 9 had incorrect contact information, 4 did not answer the phone for 3 consecutive times, and 1 developed leukemia. Ultimately, 255 study subjects were included in the statistical analysis.

### Sample characteristics

Table 1 offers specifics about the traits of the individuals. It displays the frequency, percentage, mean, standard deviation, median and quartiles. For instance, the mean age of 255 participants was (49.52±8.04) years, of which 209 (82.0%) were male and 245 (96.1%) had a spouse.

**Table 1:** 255 patients’ demographic and clinical characteristics.

Features	Total (n = 255)
Age (Years)^a^	49.52±8.04
Gender ^b^	
Male	209 (82.0)
Female	46 (18.0)
Educational level ^b^	
Primary school and below	44(17.3)
Junior high school	92 (36.1)
High school	44(17.3)
College and above	75(29.4)
Marital Status ^b^	
Have a spouse	245 (96.1)
No spouse	10 (3.9)
Job Type ^b^	
Mainly physical labor	62(24.3)
Mainly mental labor	121(47.5)
Both mental and physical labor	72(28.2)
Monthly income (yuan) ^b^	
≤5000	123(48.2)
>5000	132(51.8)
Working hours(h/d) ^b^	
≤8	139(54.5)
>8	116(45.5)
Medical Insurance (Yes)^b^	244(95.7)
Comorbidities (Yes)^b^	114(44.7)
Smoking history (Yes)^b^	176(69.0)
Drinking History (Yes)^b^	144(56.5)
Placement of stent (Yes)^b^	141(55.3)
Diagnosis	
Stable angina	38(14.9)
Acute coronary syndrome	217(85.1)
PCI Types	
Elective PCI	218(85.5)
Emergency PCI	36(14.5)
Types of drugs ^b^	
1–4	28(11.0)
5–8	170(66.7)
≥9	57(22.4)
Number of lesioned vessels ^b^	
1	84(32.9)
2	58(22.7)
≥9	113(44.3)
BMI (kg/m^2^) ^b^	
Normal(20–25kg/m^2^)	78(30.6)
Abnormal (<20 kg/m^2^ or >25 kg/m^2^)	117(69.4)
Ejection fraction (%) ^b^	
<50	85(33.3)
≥50	170(66.7)
Relapse ^b^	
Yes	37(14.5)
No	207(81.2)
No memory	11(4.3)
Length of hospital stay ^c^	6.0(5.0,7.0)
TC (mmol/L)^c^	4.04(3.40,4.87)
TG (mmol/L)^c^	1.55(1.08,2.07)
HDL-C (mmol/L)^c^	0.90(0.75,1.08)
LDL-C(mmol/L)^c^	2.27(1.71,3.21)

^a^ The mean and standard deviation are provided for the values.

^b^ The values are given as n (%).

^c^ These data are presented as median and 25th and 75th percentiles.

BMI: body mass index, EF: Ejection fraction, TC: total cholesterol, TG: triglyceride, HDL-C: high-density lipoprotein cholesterol, LDL-C: low-density lipoprotein cholesterol.

### Status of return to work, social support, return to work self-efficacy, and fatigue

In our study, return to work, which is one of the outcome variables in our study, is defined as a patient who, after being away from a previous job due to illness or injury, either goes back and resumes the former job’s duties or finds a new remunerative position. Failure to return to work after 3 months of PCI was defined as not returning to work. At the 3-month post-PCI period, 155 (60.8%) of 255 young and middle-aged patients with CHD had successfully returned to work, with a return to work time of 30 (10, 67) days. In addition to this, the social support score of 255 patients was (37.98±5.99), the self-efficacy of returning to work was (4.15±0.70), and 167 patients (65.5%) had mild fatigue. More details can be found in Table 2.

**Table 2:** Status of return to work, social support, return to work self-efficacy, and fatigue.

Features	Total (n = 255)
Return to work (Yes)^a^	155(60.8)
Time to return to work(day)^b^	30(10,67)
Social support score ^c^	37.98±5.99
Return to work self-efficacy score ^c^	4.15±0.70
Fatigue level ^a^	
Mild	167(65.5)
Moderate	62(24.3)
Severe	26(10.2)

^a^ The values are given as n (%).

^b^ These data are presented as median and 25th and 75th percentiles.

^c^ The mean and standard deviation are provided for the values.

### Relationship between general information, social support, return to work self-efficacy, fatigue and return to work

The results of univariate analysis revealed statistically significant differences in return to work among young and middle-aged patients with CHD after PCI in terms of gender, education level, age, ejection fraction, type of work, return to work self-efficacy, social support, and fatigue (all *P* < 0.05). After excluding patients who couldn’t remember whether they had recurred 3 months after PCI, we performed out a statistical analysis, which showed that relapse was not correlated to work (χ^2^ = 3.782, *P* = 0.052). We filled in the missing data using the mode-filling methodology, the results of statistical study indicated that there is no connection between the return to work and a disease relapse. The details are given in Table 3.

**Table 3:** A univariate analysis of factors affecting return to work after PCI in young and middle-aged patients with CHD (n = 255).

Variables	Return to work or not		Statistical values	*P* value^b^
	No (n = 100)	Yes (n = 155)		
Gender				
Male	72(34.4)	137(65.6)	11.040^①^	0.001**
Female	28(60.9)	18(39.1)		
Education level				
Primary school and below	21(47.7)	23(52.3)	-2.225^②^	0.026*
Junior high school	41(44.6)	51(55.4)		
High school	15(34.1)	29(65.9)		
College and above	23(30.7)	52(69.3)		
Medical insurance (Yes)	93(38.1)	151(61.9)	2.876^③^	0.168
Spouse (Yes)	95(38.8)	150(61.2)	0.508^③^	0.702
Body mass index (kg/m^2^)				
20–25	30(38.5)	48(61.5)	0.027^①^	0.780
<20 or >25	70(39.5)	107(60.5)		
Daily working hours (h)				
≤8h	40(35.7)	72(64.3)	1.027^①^	0.311
>8h	60(42.0)	83(58.0)		
Monthly income (yuan)				
≤5000	45(36.6)	78(63.4)	0.690^①^	0.406
>5000	55(41.7)	77(58.3)		
Type of work				
Strength-based	38(61.3)	24(38.7)	21.133^①^	<0.001**
Brain-based	32(26.3)	89(73.6)		
Both brain and strength	30(41.7)	42(58.3)		
Types of drugs				
1–4	11(39.3)	17(60.7)	-0.387^②^	0.699
5–8	65(38.2)	105(61.8)		
≥9	24(42.1)	33(57.9)		
Number of diseased vessels				
1	33(39.3)	51(60.7)	-0.102^②^	0.919
2	22(37.9)	36(62.1)		
≥3	45(39.8)	68(60.2)		
Ejection fraction (%)				
<50	46(51.4)	39(45.9)	6.269^①^	0.001**
≥50	54(31.8)	116(68.2)		
Diagnosis				
Stable angina	10(26.3)	28(73.7)	3.117^①^	0.077
Acute coronary syndrome	90(41.5)	127(58.5)		
PCI Types				
Elective PCI	81(37.2)	137(62.8)	2.045^①^	0.153
Emergency PCI	19(51.4)	18(48.6)		
Relapse (Yes)	19(51.4)	18(48.6)	2.674^①^	0.102
Stent implantation (Yes)	59(41.8)	82(58.2)	0.914^①^	0.339
Comorbidities (Yes)	49(43.0)	65(57.0)	1.277^①^	0.268
Fatigue level				
Mild	54(32.3)	113(67.7)	-3.804^②^	<0.001**
Moderate	24(38.7)	38(61.3)		
Severe	22(84.6)	4(15.4)		
Age (years)	51.46±7.99	48.27±7.84	3.136^④^	0.002**
Return to work self-efficacy	3.90±0.63	4.31±0.69	-4.910^④^	<0.001**
Social support	35.66±6.39	39.48±5.21	-5.008^④^	<0.001**
Length of hospital stay	6.00(5.00,7.00)	6.00(5.00,6.00)	-1.853^⑤^	0.064
TC (mmol/L)	4.02(3.47,4.70)	4.08(3.35,4.89)	-0.155^⑤^	0.877
TG (mmol/L)	1.48(1.03,1.82)	1.63(1.14,2.24)	-1.749^⑤^	0.080
HDL-C(mmol/L)	0.93(0.75,1.13)	0.89(0.78,1.06)	-0.222^⑤^	0.824
LDL-C(mmol/L)	2.24(1.67,3.37)	2.38(1.75,3.20)	-0.491^⑤^	0.623

^①^The effect factors of the return to work group with those of the non-return to work group were compared using the chi-square test.

^②^Wilcoxon test was used to compare the factors that may influence getting back to work.

^③^The Fisher’s exact test was utilized to contrast factors influencing return to work.

^④^An independent sample t-test was applied to make a comparison of the factors affecting return to work.

^⑤^The Mann-Whitney U test was conducted to analyze the factors that influence return to work.

TC: total cholesterol, TG: triglyceride, HDL-C: high-density lipoprotein cholesterol, LDL-C: Low-density lipoprotein cholesterol.

*P< 0.05

**P< 0.01

The dependent variable in this study was returning to work or not, and we used the statistically significant variables from the univariate analysis (gender, education level, age, ejection fraction, type of work, self-efficacy of returning to work, social support, and fatigue) as independent variables and used multivariable logistic regression analysis to examine the data, and the results of the statistical analysis showed that gender, ejection fraction, self-efficacy of returning to work, fatigue, social support, and type of work were the independent influencing factors for returning to work after PCI (All *P*<0.05). Table 4 displays further information about the findings.

**Table 4:** Multivariable logistic regression of the factors influencing return to work (n = 255).

Items	B	Exp(B)	95%CI	*P* value
Age	-0.038	0.963	(0.923,1.005)	0.081
Education level				
Primary school and below (reference)				0.276
Junior high school	-0.518	0.596	(0.242,1.463)	0.259
High school	0.390	1.476	(0.499,4.366)	0.481
College and above	-0.299	0.742	(0.270,2.033)	0.561
Gender (Female)	-0.970	0.379	(0.169,0.851)	0.019*
Ejection fraction (≥50%)	0.719	2.053	(1.085,3.885)	0.027*
Social support	0.059	1.060	(1.003, 1.121)	0.040*
Return to work self-efficacy	0.638	1.893	(1.140,3.144)	0.014*
Type of work				
Strength-based (reference)				0.013*
Brain-based	1.065	2.902	(1.361,6.190)	0.006**
Both brain and strength	1.053	2.867	(1.224,6.715)	0.015*
Level of fatigue				
Severe (reference)				0.029*
Moderate	1.796	6.023	(1.596,22.725)	0.008**
Mild	1.395	4.035	(1.104,14.751)	0.035*

*P< 0.05

**P< 0.01

## Discussion

Return to work means that the patient returns to work and continues to undertake work tasks after leaving the workplace due to injury or illness. According to our study, 255 young and middle-aged patients with CHD returned to their job at a rate of 60.8% at 3 months following PCI, which is lower than the data from a study conducted by Mehrdad at Tehran Heart Center [31]. The study, which surveyed 226 patients with CHD who underwent coronary artery bypass grafting, reported that 155 (68.9%) patients returned to work at 3 months post-operatively [31]. Consider the probable reason that nearly half of the cases in this study were treated with medication only, and there was a possibility of recurrence of coronary heart disease symptoms such as chest pain and tightness, thus impairing return to work. On the other hand, the patients treated by coronary artery bypass graft have more obvious improvement of symptoms such as chest pain and stuffiness, and the mortality rate, myocardial infarction rate and re-vascularization rate were lower than those treated by PCI [32], so the return to work rate was relatively elevated. This study was conducted during the COVID-19 epidemic. Because of the epidemic, some work units have closed down, layoffs, etc., and many people have lost their jobs. When they lose their jobs, it is a challenging problem for them to obtain another position with a source of income. Also, some people infected with the novel coronavirus recover more slowly than uninfected patients, and whose prognosis is generally terrible, which makes it extremely hard for them to return to work [33].

However, the rate of return to work at 3 months after PCI in young patients with CHD in this study was higher than in the study by Nascimento [34], which investigated 117 stroke patients from four hospitals prospectively over a six-month period and found that only 52 (44%) returned to work within 6 months of discharge. The reason is that a certain proportion of stroke patients have sequelae (such as hemiplegia, aphasia, etc.) after the disease, which prevents them from returning to work [35]. Studies have pointed out that patients who have returned to work have better quality of life, spiritual and psychological status, cost of living, and level of family gain than those who have not returned to work [9, 10]. So, it is therefore necessary to promote the return to work of patients with CHD who are of employment age.

Nevertheless, return to work is a dynamic process. One study reported that return to work rates in patients with CHD did not increase with time, but rather showed a decreasing trend [17]. Another study noted that although some patients with myocardial infarction had returned to work, their experience of working after the disease was not favorable, and they were stuck coping with the disease and work with a higher level of health requirements [36]. Returning to work is not only important to minimize the cost of living, but is also an important means to achieve self-worth for young and middle-aged patients, and is a crucial aim for cardiac rehabilitation and secondary prevention [37]. Because of this, it is critical to act with a focus on sustaining employment to support a better return to work in patients with CHD after PCI.

According to the study’s findings, gender was an independent variable in how many young and middle-aged patients with coronary heart disease returned to work following PCI (0.379 times more female patients than male patients did), which is in line with the conclusions of Smedegaard [21]. Because men typically serve as the major breadwinners in traditional Chinese culture, their return to work rate is higher than that of women. One study also revealed that men had a higher level of mental and psychological well-being and physical health than women do during the disease recovery process, which raises their likelihood of achieving a successful return to work [38]. So, clinical professionals need to focus more on female patients and help them return to work successfully as soon as possible.

In accordance with the results of Mirmohammadi [39], the results of this study showed that the return to work rate was 2.053 times higher in individuals with ejection fraction ≥ 50% than in patients with ejection fraction < 50%. Ejection fraction, one of the markers of cardiac function, is the ratio of cardiac output per beat to the ventricle’s end-diastolic volume, reflecting the systolic function of the heart. Ejection fraction ≥50% indicates normal cardiac systolic function. An ejection fraction of <50% implies that the patient has abnormal cardiac systolic function, and an ejection fraction of ≤40% is even more indicative of cardiac systolic dysfunction, where the blood pumped by the heart cannot fully meet the physiological demands of the body to cope with work, thus preventing return to work [40].

According to our study, patients who did mainly mental work had a 2.902 greater probability of returning to work than those who did mainly physical labor (95%CI: 1.361–6.190, *P* = 0.006). In addition, the rate of return to work was 2.867 times more for patients with both mental and physical strength than for those with predominantly physical work (95%CI: 1.224–6.715, *P* = 0.015). As it is shown in Table 4. Firstly, patients who work primarily in manual labor have the lowest rates of returning to their occupations due to the demanding physical nature of their profession and the necessity to refrain from intensive activity after discharge due to the limitations of the illness. Secondly, the majority of those patients who work primarily as physical laborers also have poor levels of education, which restricts their employment alternatives and increases the chances that they won’t be able to return to work.

Return to work self-efficacy refers to a patient’s perceptions of their competence to adopt behaviors and accomplish goals (a job or a particular task). Similar to the findings of Black [41], our study’s findings showed that individuals with higher return-to-work self-efficacy were more likely to return to work, with each unit increase in the score being related with an 89% increase in the likelihood of returning to employment. Since patients who seem to have a high level of return-to-work self-efficacy are more likely to cope with their illness positively, to re-establish their social role, and to return to work earlier [42].

Additionally, our research found that patients with mild fatigue returned to their job at a rate that was 3.035 times higher than patients with severe fatigue (95%CI: 1.104–14.751, *P* = 0.035), and patients with moderate fatigue returned to work at a rate that was 6.023 times greater than patients with severe fatigue (95%CI: 1.596–22.725, *P* = 0.008), which is comparable to the research by Rutkowsk [43]. Fatigue is a sensation of physical and mental unwellness, mainly showing as a subjective feeling of tiredness, physical muscle weakness, memory impairment, etc. Fatigue can raise the risk of major adverse cardiovascular events such angina pectoris, myocardial infarction, and unplanned revascularization, which can prevent employees from returning to their workplaces [44]. Also, continuous fatigue can result in uncomfortable emotions like anxiety, depression, and anger, which weakens one’s capacity to return to work and prevents patients from successfully returning to the workforce [45].

Our study confirmed that social support is a protective factor for young and middle-aged patients to return to work post PCI; that is, the more social support a patient has, the easier it is for them to do so, which is similar to Kamp’s study’s findings [46]. Social support is the concept for the material, emotional, or spiritual support that people receive via social networks. A good social support can offer both substance and moral support to patients, which is conducive to their physical and mental health, improve their social adjustment ability, and thus facilitate their smooth return to work [47]. Our study showed that patients who had returned to work had a significantly higher social support score (39.48±5.21) than those who had not returned to work (35.66±6.39). The young and middle-aged group, however, leads a fast-paced existence, experiences significant work-related stress, and has a relatively small social network. We must educate patients and their families on the need of fostering a supportive social environment so that patients can successfully manage their condition and quickly return to work.

In contrast to the findings of Sun [48], our study revealed that age is not an independent factor for return to employment. The subjects of this study [48] were only young and middle-aged people, with an age of (49.52 ± 8.04) years old. In the Sun′s study, 91 participants (67%) were between the ages of 60 and 80, and only 45 cases (33%) were younger than the age of 49. In our nation, the mandatory retirement age is 60 for men and 55 for women. The overwhelming majority of people choose to retire in order to enjoy their old age and the happiness of heaven, which may account for the conflicting results of our research. Yet, because our study population was obtained from a Class iii Grade A hospital in Wuxi, one of China’s richest cities, they might not have an immediate need for a job right away. Additionally, our study was in the epidemic period of COVID-19, so that those patients closer to the retirement age are more likely to choose not to return to work or wait for the physical and working environment to ease before returning to work.

In accordance with the findings of the majority of studies [22, 49], our findings also indicated that educational attainment was not a standalone determinant for return to employment. Their study revealed that education level is associated with going back to work in the univariate analysis, but in a regression analysis, education level was left out of the regression model, which is consistent with our study’s findings. The possible reason is that the new coronavirus epidemic is currently facing a severe employment situation. Regardless of the educational level of patients, their work is affected to a certain extent by the COVID-19. At this time, the possibility of returning to work may be related to more personal psychological status and willingness [50, 51].

## Conclusion

To our knowledge, this is the first study to be conducted in China that looks at the variables influencing return to work following a PCI in people who are young and middle-aged with CHD. Our study demonstrates the need to improve the current situation for young and middle-aged individuals with CHD returning to work after PCI. Ejection fraction, gender, fatigue, social support, kind of work, and return-to-work self-efficacy were the independent influences on return-to-work in young and middle-aged patients after PCI. Our research suggests that in order to successfully assist patients in returning to work, health-care workers can design personalized multidimensional treatment programs for them, taking into account their health condition, body function, and contextual factors.

These disadvantages remain exist with this investigation, though. First, the process of going back to work is dynamic, and some people end up leaving their previous occupations after doing so. The current state of returning to the workforce at various time points and its affecting elements require longitudinal surveys to be conducted in future. The outcome variable in this study is whether or not to return to work, however state variables can also be used to assess going back to work. In follow-up studies, the time to return to work, the ability to return to work, and the ability to adapt after returning to work need to be explored in many aspects, so as to benefit many patients. Second, the study did not take into consideration variables like the work environment, job satisfaction, and employers that could have an impact on a person’s decision to return to the workforce. Future research might take into account this aspect of the influencing elements to comprehend the factors that affect patients’ ability to return to work in a more thorough and comprehensive manner. Finally, because of time and resource limitations, the sample for this study was drawn from just one tertiary care hospital in Wuxi, which could result in biased or poorly represented study results. To confirm the findings of this study, it is recommended that future studies perform multicenter clinical trials and utilize better sampling techniques.

## Supporting information

S1 File Protocol. (DOCX)

S2 File Files of data. (XLS)

S3 File STROBE-checklist. (DOCX)

## References

[1] China Cardiovascular Health and Disease Report Writing Group. Report on cardiovascular Health and diseases burden in China: an updated Summary of 2020.Chinese Circulation Journal.2021;36(6):521–545.

[2] Writing Group Members, Mozaffarian D, Benjamin EJ, Go AS, Arnett DK, Blaha MJ, et al. Heart disease and stroke statistics-2016 update: A report from the American Heart Association.Circulation.2016;133(4):e38–e360. doi: 10.1161/CIR.0000000000000350 26673558

[3] Arora S, Stouffer GA, Kucharska-Newton AM, Qamar A, Vaduganathan M, Pandey A, et al. Twenty year trends and sex differences in young adults hospitalized with acute myocardial infarction.Circulation.2019;139(8):1047–1056. doi: 10.1161/CIRCULATIONAHA.118.037137 30586725 PMC6380926

[4] Liu J, Zhao D, Liu J, Qi Y, Sun JY, Wang Y, et al. Changes in the diagnosis and treatment of hospitalized patients with acute coronary syndrome from 2006 to 2012 in China.Chinese Journal of Cardiology.2014;42(11):957–962. doi: 10.3760/cma.j.issn.0253-3758.2014.11.016 25620260

[5] Hoole SP, Bambrough P. Recent advances in percutaneous coronary intervention.Heart.2020;106(18):1380–1386. doi: 10.1136/heartjnl-2019-315707 32522821

[6] Liang F, Hu DY, Fang W, Shen ZJ. Reperfusion therapy for acute ST-segment elevation myocardial infarction.Chin J Evid Based Cardiovasc Med.2019;11(3):263–274.

[7] Du RF, Chen CY. A qualitative study on psychological experience of patients with myocardial infarction after returning to work.Chin J Nurs.2018;53(8):920–925. doi: 10.3761/j.issn.0254-1769.2018.08.004

[8] Guo YW, Fu B, Mei YX, Lin BL, Zhang ZX. Advance in measurement instruments of return-to-work (review).Chinese Journal of Rehabilitation Theory and Practice.2018;24(12):1417–1421. doi: 10.3969/j.issn.1006-9771.2018.12.012

[9] Cauter JV, Bacquer D, Clays E, Smedt D, Kotseva K, Braeckman L. Return to work and associations with psychosocial well-being and health-related quality of life in coronary heart disease patients:Results from EUROASPIRE IV.Eur J Prev Cardiol.2019;26(13):1386–1395. doi: 10.1177/2047487319843079 30971121

[10] Salzwedel A, Koran I, Langheim E, Schlitt A, Nothroff J, Bongarth C, et al. Patient-reported outcomes predict return to work and health-related quality of life six months after cardiac rehabilitation: Results from a German multi-centre registry (OutCaRe).PLoS One.2020;15(5):e0232752. doi: 10.1371/journal.pone.0232752 32369514 PMC7199966

[11] Olsen SJ, Schirmer H, Wilsgaard T, Bønaa KH, Hanssen TA. Employment status three years after percutaneous coronary intervention and predictors for being employed:A nationwide prospective cohort study.Eur J Cardiovasc Nurs.2020;19(5):433–439. doi: 10.1177/1474515120903614 32106706

[12] Wang H, Lin P, Tao H, Shi LF, Xue YR. The influence factors of the long-term social functioning in patients after percutaneous coronary intervention.Chinese Journal of Nursing.2015;50(3):345–349. doi: 10.3761/j.issn.0254-1769.2015.03.022

[13] Jiang Z, Dreyer RP, Spertus JA, Masoudi FA, Li J, Zheng X, et al. Factors associated with return to work after acute myocardial infarction in China.JAMA Netw Open.2018;1(7):e184831. doi: 10.1001/jamanetworkopen.2018.4831 30646375 PMC6324382

[14] Reibis R, Salzwedel A, Abreu A, Corra U, Davos C, Doehner W, et al. The importance of return to work: How to achieve optimal reintegration in ACS patients[J].Eur J Prev Cardiol.2019;26(13):1358–1369. doi: 10.1177/2047487319839263 30971111

[15] Warraich HJ, Kaltenbach LA, Fonarow GC, Peterson ED, Wang TY. Adverse change in employment status after acute myocardial infarction: analysis from the TRANSLATE-ACS study.Circ Cardiovasc Qual Outcomes.2018;11(6):e004528. doi: 10.1161/CIRCOUTCOMES.117.004528 29895612 PMC6003623

[16] Stendardo M, Bonci M, Casillo V, Miglio R, Giovannini G, Nardini M, et al. Predicting return to work after acute myocardial infarction:Socio-occupational factors overcome clinical conditions.PLoS One.2018;13(12):e0208842. doi: 10.1371/journal.pone.0208842 30543689 PMC6292571

[17] Worcester MU, Elliott PC, Turner A, Pereira JJ, Murphy BM, Le Grande MR, et al. Resumption of work after acute coronary syndrome or coronary artery bypass graft surgery.Heart Lung Circ.2014;23(5):444–453. doi: 10.1016/j.hlc.2013.10.093 24309233

[18] Hu YY, Mao FY, Zhang J, Yu L, Wu Q. Influencing factors of return to work among young and middle-aged patients with coronary heart disease.Journal of Nursing Science.2022;37(15):20–23.

[19] Xie C, Wang Y, Xu Y, Li LS, Chang WH, Gao Q, et al. Status and influencing factors of returning to work in premature coronary patients with interventional therapy.Henan Medical Research,2022,31(12):2156–2162. doi: 10.3969/j.issn.1004-437X.2022.12.010

[20] Chen B. Sample size methodology for multivariate analysis-synthetic estimate method for sample size in multivariate analysis. Injury Medicine.2012;1(4):58–60.

[21] Smedegaard L, Numé AK, Charlot M, Kragholm K, Gislason G, Hansen PR. Return to work and risk of subsequent detachment from employment after myocardial infarction:insights from Danish nationwide registries.J Am Heart Assoc.2017;6(10):e006486. doi: 10.1161/JAHA.117.006486 28978528 PMC5721858

[22] Yang SS, Liu JE, Su YL, Zhao Y, Liu YF. The investigation on the status and influence factors of returning to work among breast cancer survivors.Chinese Nursing Management.2020;20(6):821–825.

[23] Wang J, Qin HY, Huang ZY, Fan YY, Hu W. Research progress on the status and influencing factors of returning to work in survivors of nasopharyngeal cancer.China Health Standard Management.2022;13(14):194–198.

[24] Biering K, Lund T, Andersen JH, et al. Effect of psychosocial work environment on sickness absence among patients treated for ischemic heart disease.J Occup Rehabil.2015;25(4):776–782. doi: 10.1007/s10926-015-9587-0 26077204

[25] Mendoza TR, Wang XS, Cleeland CS, Morrissey M, Johnson BA, Wendt JK, et al. The rapid assessment of fatigue severity in cancer patients: use of the Brief Fatigue Inventory.Cancer.1999;85(5):1186–1196. doi: 10.1002/(sici)1097-0142(19990301)85:5&lt;1186::aid-cncr24&gt;3.0.co;2-n 10091805

[26] Gao LP, Zhu XQ, Zhao H, Jiao HM, Chen DX. Studies on internal consistency and test-retest reliability of brief fatigue inventory in cancer patients.Nurs J Chin PLA.2009;26(8):1–3.

[27] Lagerveld S E, Blonk R W B, Brenninkmeijer V, Schaufeli Wilmar B. Return to work among employees with mental health problems:development and validation of a self-efficacy questionnaire. Work & Stress.2010;24(4):359–375. doi: 10.1080/02678373.2010.532644

[28] Gao YX, Qu QR, Wang BX, Zang KX, Cui TJ. Chinese translation of the return-to-work self-efficacy questionnaire in cancer patients andults reliability and validity test.Nurs J Chin PLA.2021;38(7):52–55.

[29] Xiao SY. Theoretical basis and research application of the social support rating scale.Journal of Clinical Psychiatry.1994;(2):98–100.

[30] Zhang AH, Song J. Effects of social support and impact of event on posttraumatic growth of patients after percutaneous coronary intervention.Chinese Journal of Practical Nursing.2018;34(2):88–93.

[31] Mehrdad R, Ghadiri Asli N, Pouryaghoub G, Saraei M, Salimi F, Nejatian M. Predictors of early return to work after a coronary artery bypass graft surgery (CABG).Int J Occup Med Environ Health.2016;29(6):947–957. doi: 10.13075/ijomeh.1896.00798 27869245

[32] Wang H, Wang H, Wei Y, Li X, Jhummun V, Ahmed MA. Ten-year outcomes of percutaneous coronary intervention versus coronary artery bypass ggrafting for patients with type 2 diabetes mellitus suffering from left main coronary disease: A meta-analysis.Diabetes Ther.2021;12(4):1041–1054. doi: 10.1007/s13300-021-01025-x 33641081 PMC7994472

[33] Godeau D, Petit A, Richard I, Roquelaure Y, Descatha A. Return-to-work, disabilities and occupational health in the age of COVID-19.Scand J Work Environ Health.2021;47(5):408–409. doi: 10.5271/sjweh.3960 34003294 PMC8259700

[34] Nascimento LR, Scianni AA, Ada L, Fantauzzi MO, Hirochi TL, Teixeira-Salmela LF. Predictors of return to work after stroke:a prospective, observational cohort study with 6 months follow-up. Disabil Rehabil.2021;43(4):525–529. doi: 10.1080/09638288.2019.1631396 31242399

[35] Guzik A, Kwolek A, Drużbicki M, Przysada G. Return to work after stroke and related factors in Poland and abroad:A literature review.Work.2020;65(2):447–462. doi: 10.3233/WOR-203097 31985482

[36] Du RF, Wang PP, Cheng CY. Investigation of health needs and analysis of influencing factors in patients who have returned to work after myocardial infarction.Chinese General Practice.2019;22(5):586–590.

[37] Cardiovascular disease committee of China society of rehabilitation medicine.Guidelines for cardiovascular rehabilitation and secondary prevention in China 2018 simplified edition.Chinese Journal of Internal Medicine.2018;57(11):802–810. doi: 10.3760/cma.j.issn.0578-1426.2018.11.003 30392235

[38] Tang XF, Song Y, Xu JJ, Wang HH, Jiang L, Jiang P. Clinical characteristics and prognosis between male and female patients with premature coronary artery disease after intervention.Chinese Journal of Cardiology,2019;47(10):798–805. doi: 10.3760/cma.j.issn.0253-3758.2019.10.006 31648462

[39] Mirmohammadi SJ, Sadr-Bafghi SM, Mehrparvar AH, Gharavi M, Davari MH, Bahaloo M, et al.Evaluation of the return to work and its duration after myocardial infarction.ARYA Atheroscler.2014;10(3):137–140. 25161683 PMC4144381

[40] You LM, Wu Y. Internal Medicine Nursing (7th Edition).People’s Medical Publishing House,2017:160.

[41] Black O, Sim MR, Collie A, Smith P. Differences over time in the prognostic effect of return to work self-efficacy on a sustained return to work.J Occup Rehabil.2019;29(3):660–667. doi: 10.1007/s10926-018-09824-z 30719610

[42] Volker D, Zijlstra-Vlasveld MC, Brouwers EP, van Lomwel AG, van der Feltz-Cornelis CM. Return-to-work self-efficacy and actual return to work among long-term sick-listed employees.J Occup Rehabil.2015;25(2):423–31. doi: 10.1007/s10926-014-9552-3 25354750

[43] Rutkowski NA, Sabri E, Yang C. Post-stroke fatigue:A factor associated with inability to return to work in patients <60 years-A 1-year follow-up. PLoS One. 2021;16(8):e0255538. doi: 10.1371/journal.pone.0255538 34347804 PMC8336834

[44] O’Keefe-McCarthy S, Ready L. Impact of prodromal symptoms on future adverse cardiac-related events: a Systematic review. J Cardiovasc Nurs.2016;31(1):E1–E10. doi: 10.1097/JCN.0000000000000207 25419940

[45] Carlsen K, Jensen AJ, Rugulies R, Christensen J, Bidstrup PE, Johansen C, et al. Self-reported work ability in long-term breast cancer survivors.A population-based questionnaire study in Denmark. Acta Oncol.2013;52(2):423–9. doi: 10.3109/0284186X.2012.744877 23282112

[46] Kamp T, Stevens M, Van Beveren J, Rijk PC, Brouwer R, Bulstra S, et al. Influence of social support on return to work after total hip or total knee arthroplasty: a prospective multicentre cohort study. BMJ Open.2022;12(5):e059225. doi: 10.1136/bmjopen-2021-059225 35623752 PMC9150170

[47] Guo XL, Yin HY. The Intermediary role of positive coping style in the relationship between social support and mental health of pediatric nurses. Nurs J Chin PLA.2020,37(3):50–53. doi: 10.3969/j.issn.1008-9993.2020.03.014

[48] Sun W, Gholizadeh L, Perry L, Kang K. Predicting return to work following myocardial infarction: a prospective longitudinal cohort study. Int J Environ Res Public Health.2022;19(13):8032. doi: 10.3390/ijerph19138032 35805690 PMC9266191

[49] Zhong ZJ, Hu SZ. Investigation on adaptability of return to work among patients with hepatocellular carcinoma after radical resection[J]. Mil Nurs.2023;40(1):27–30.

[50] Giorgi G, Lecca LI, Alessio F, Finstad GL, Bondanini G, Lulli LG, et al. COVID-19-related mental health effects in the workplace:a narrative review.Int J Environ Res Public Health.2020;17(21):7857. doi: 10.3390/ijerph17217857 33120930 PMC7663773

[51] Garzillo EM, Cioffi A, Carta A, Monaco MGL. Returning to work after the COVID-19 pandemic earthquake:a systematic review.Int J Environ Res Public Health.2022;19(8):4538. doi: 10.3390/ijerph19084538 35457407 PMC9024882

