# Supplementary material for: Factors influencing return to work 3 months after percutaneous coronary intervention in young and middle-aged patients with coronary heart disease: A single-center, cross-sectional study
Source: PLoS One. 2023 Apr 19;18(4):e0284100. doi: 10.1371/journal.pone.0284100 (PMC10115307; doi:10.1371/journal.pone.0284100)
Supplement: S1 File — (DOCX) [file pone.0284100.s001.docx]

**1 Background**

Coronary artery atherosclerotic heart disease or coronary heart disease(CHD), myocardial ischemia and hypoxia caused by narrowing or blockage of coronary arteries. With high morbidity, mortality and young age[1]. As reported by the American Heart Association[2], by 2030, it is projected that more than 23.6 million people will die from cardiovascular disease worldwide and the economic burden of cardiovascular disease will exceed $1 trillion.Arora et al.[3] noted that, from 1995 to 2014, the proportion of young CHD patients hospitalized in the United States increased each year.Chinese scholars Liu Jun et al.[4] reported that the mean age of patients with CHD in 2012 in China was less than the mean patient age in 2006, and there was a 32.4% increase in young and middle-aged CHD patients in 2006. Not only that, Sun Wenhui[5] reported that the incidence rate of coronary heart disease among young and middle-aged people in Zhengzhou was 33.45/100,000 and the mortality rate was 14.68/100,000 from 2015 to 2016, showing an increasing trend.Summary of China Cardiovascular Health and Disease Report 2020 [1] stated that the prevalence of cardiovascular disease in China continues to rise, with the current number of CHD patients at 11.39 million, and is the leading cause of death among urban and rural residents in China. The total cost of hospitalization for cardiovascular disease is also increasing rapidly, with an average annual growth rate much higher than the growth rate of GNP over the last decade or so. Therefore, it is extremely necessary to actively prevent and treat CHD in young and middle-aged people.

Percutaneous coronary intervention (PCI) is a treatment that uses catheter technology based on coronary angiography to enlarge the internal diameter of narrowed coronary arteries by means of balloon pressure dilation, mechanical cutting, laser vaporization, and placement of metal stents to improve myocardial blood supply and relieve patients' clinical symptoms. It is now the main treatment for cardiovascular diseases because of its advantages such as less trauma and faster recovery. Data show that the total number of coronary interventions in mainland China alone was 915,256 cases in 2018, and the average number of cases per million population in China was 651 in 2018, with an average of 1.46 stents placed in patients with CHD[1]. The prognosis of the increasingly large group of patients after PCI warrants our focused attention.Since young and middle-aged people shoulder important social and family responsibilities, and are the main group of social wealth creation, as well as the economic and spiritual pillar of the family.Increasingly, patients are hoping to return to work and regain some social role after treatment.

Returning to work means that patients return to their former jobs or accept new paid jobs[6], which is important for improving patients' quality of life, reducing negative emotions and achieving self-worth, and is also one of the important indicators of disease recovery[7-8]. A foreign study of 3,291 patients in 24 European countries found that 76% of patients could return to work between 6 months and 3 years after discharge from hospital[9]. A prospective cohort study of 3-year employment status of patients after PCI by Norwegian scholars[10] found that approximately 89% of patients returned to work. Wang Hui conducted a questionnaire survey of 428 patients 3 years after PCI and showed that 5.7% of patients were unable to work or had serious health problems at work, and 58.8% had a significant decrease in work level and needed to reduce their work[11]. Jiang et al.[12] found that 55.9% of patients returned to work within 1 year after acute myocardial infarction in a prospective cohort study of 1165 patients treated with PCI in 53 hospitals in 21 provinces across China. Compared with the survey data on return to work in foreign countries, the return to work rate of patients with severe coronary heart disease in China is relatively low, and a variety of problems can occur after returning to work.Therefore, it is necessary to understand the factors influencing the return to work of our young and middle-aged patients with coronary artery disease to facilitate their early return to work.

However, return to work is a complex and dynamic process that involves physical, psychological, social and occupational factors. Moreover, few studies have been reported on the factors influencing return to work after PCI in young and middle-aged patients with coronary artery disease in China. Therefore, the aim of this study was to explore the factors influencing the return to work after PCI in China patients with CHD, and to provide a reference basis for constructing a return to work intervention program for young and middle-aged patients with coronary heart disease to promote their early return to society and family.

**2 Purpose and significance of the study**

To understand the current status of return to work after PCI in young and middle-aged patients with CHD and to explore the factors affecting return to work, and to provide a theoretical and reference basis for the construction of a return to work intervention program to promote the early return of patients to society and their families.

**3 Definition of concepts**

3.1 Coronary Heart Disease

It refers to heart disease caused by atherosclerosis of the coronary arteries narrowing or blocking the lumen, or (and) myocardial ischemia and hypoxia or necrosis due to functional changes (spasm) in the coronary arteries, collectively known as coronary heart disease , or coronary artery disease[13].

3.2 Return to Work

In this study it is defined as performing work with pay prior to hospitalization and returning to the original job or accepting a new position with a salary after discharge[6].

**References**

[1] China Cardiovascular Health and Disease Report Writing Group. Report on Cardiovascular Health and Diseases Burden in China: an Updated Summary of 2020.Chinese Circulation Journal.2021;36(6):521-545.doi:10.3969/j.issn.1000-3614.2021.06.001.

[2] Writing Group Members,Mozaffarian D,Benjamin EJ,Go AS,Arnett DK,Blaha MJ,Cushman M,et al.Heart Disease and Stroke Statistics-2016 Update: A Report From the American Heart Association.Circulation.2016;133(4):e38-e360.doi:10.1161/CIR.0000000000000350.

[3] Arora S,Stouffer GA,Kucharska-Newton AM,Qamar A,Vaduganathan M,Pandey A,et al.Twenty Year Trends and Sex Differences in Young Adults Hospitalized With Acute Myocardial Infarction.Circulation.2019;139(8):1047-1056.doi:10.1161/CIRCULATIONAHA.118.037137.

[4] Liu J,Zhao D,Liu J, Qi Y,Sun JY,Wang Y,et al.Changes in the diagnosis and treatment of hospitalized patients with acute coronary syndrome from 2006 to 2012 in China.Chinese Journal of Cardiology.2014;42(11):957-962.doi:10.3760/cma.j.issn.0253-3758.2014.11.016.

[5] Sun WH,Li JB,Feng HF,Chang ZJ.Analysis of the monitoring data for acute coronary disease cases of Zhengzhou residents between 2015 and 2016.Modern Preventive Medicine.2018;45(18):3422-3424+3428.

[6] Guo YW,Fu B,Mei YX,Lin BL,Zhang ZX.Advance in Measurement Instruments of Return-to-work (review).Chinese Journal of Rehabilitation Theory and Practice.2018;24(12):1417-1421.doi:10.3969/j.issn.1006-9771.2018.12.012.

[7] Cauter JV,Bacquer D,Clays E,Smedt D,Kotseva K,Braeckman L.Return to work and associations with psychosocial well-being and health-related quality of life in coronary heart disease patients:Results from EUROASPIRE IV.Eur J Prev Cardiol.2019;26(13):1386-1395.doi:10.1177/2047487319843079.

[8] Salzwedel A,Koran I,Langheim E,Schlitt A,Nothroff J,Bongarth C,et al.Patient-reported outcomes predict return to work and health-related quality of life six months after cardiac rehabilitation: Results from a German multi-centre registry (OutCaRe).PLoS One.2020;15(5):e0232752.doi:10.1371/journal.pone.0232752.

[9] Olsen SJ,Schirmer H,Wilsgaard T,Bønaa KH,Hanssen TA.Employment status three years after percutaneous coronary intervention and predictors for being employed:A nationwide prospective cohort study.Eur J Cardiovasc Nurs.2020;19(5):433-439.doi:10.1177/1474515120903614.

[10] Wang H,Lin P,Tao H,Shi LF,Xue YR.The influence factors of the long-term social functioning in patients after percutaneous coronary intervention.Chinese Journal of Nursing.2015;50(3):345-349.doi:10.3761/j.issn.0254-1769.2015.03.022.

[11] Jiang Z,Dreyer RP,Spertus JA,Masoudi FA,Li J,Zheng X,et al.Factors Associated With Return to Work After Acute Myocardial Infarction in China.JAMA Netw Open.2018;1(7):e184831.doi:10.1001/jamanetworkopen.2018.4831.

[12] Chen B.Sample Size Methodology for Multivariate Analysis-Synthetic Estimate Method for Sample Size in Multivariate Analysis.Injury Medicine.2012;1(4):58-60.doi:10.3868/j.issn.2095-1566.2012.04.012.

[13] Ge Junbo, Xu Yongjian, Wang Chen. Internal medicine (9th ed.) [M]. People's Health Publishing House, 2018.

**4 Research Subjects and Methods**

4.1 Subjects

Taking a convenient sampling method.Patients with coronary heart disease who received treatment at the Department of Cardiology, Affiliated Hospital of Jiangnan University from December 2020 to August 2021 were selected as study subjects. The recruitment of the first case 1 subjects was planned for December 30, 2020. Inclusion criteria: ① age 18-60; ② first diagnosis of coronary artery disease and treatment with PCI; ③ had relatively stable salary job before illness; ④ willing to participate in this study and sign the informed consent form. Exclusion criteria: ① have psychiatric or cognitive impairment that makes it difficult to cooperate; ② have a serious disease that affects survival, such as malignancy, advanced liver or kidney disease; ③ those who are unable or refuse to complete the follow-up.

**4.2 Sample Size**

According to the sample size requirement of multi-factor regression analysis [14], the sample size is the principle of being 5-10 times the number of variables, there are 22 influential variables in this study, and the required sample size was calculated to be 110-220 cases.Taking into account a 20% sample attrition rate and other unpredictable factors, a final sample size of 280 cases was drawn up for this study.

**4.3 Research tools**

4.3.1 General information questionnaire

The questionnaire was designed by the research team based on a review of the literature and included mainly demographic and clinical information. The demographic information included: age, gender, education, monthly income, payment method, history of alcohol consumption, and history of smoking. Clinical information included: stent implantation or not, left ventricular ejection fraction, ultrasensitive C-reactive protein, total cholesterol, triglycerides, HDL cholesterol, LDL cholesterol, et al.

4.3.2 The Chinese Version of the Brief Fatigue Inventory (BFI-C)

The scale was developed by Mendoza et al. and consists of 9 entries. The first 3 entries assess the current level of fatigue, the general level and the worst level of fatigue in the last 24 h. Entries 4-9 assess the impact of fatigue on different aspects of life. The assessment was performed using the 0-10 line method, with 0 indicating none and 10 indicating the most severe. the mean score of the 10 items was the final score of the scale. Fatigue was classified into 3 levels based on the scores, with 1-3 being mild fatigue, 4-6 being moderate fatigue, and 7-10 being severe fatigue. The Cronbach's α coefficient of the Chinese version of the scale was 0.94[15].

4.3.3 The Social Support Rating Scale(SSRS)

The scale was developed by Xiao to measure the current status of patients' social support, with 10 items and 3 dimensions: subjective support, objective support, and support utilization[16] . The total score of the scale ranges from 12 to 66, with higher scores indicating better social support.

4.3.4 The Return to Work Self-efficacy Questionnaire

The questionnaire was administered to measure patients' return to work self-efficacy. The 11-item questionnaire uses a Likert 6-point scale (1 to 6) and asks study participants to rate their statements about their job assuming they return to work tomorrow. The final score on the scale was the mean of the 11 items, with higher scores denoting higher levels of return-to-work self-efficacy. For the Chinese translation of RTW-SE, the Cronbach's α coefficient was 0.93[17].

**4.4 Data Collection**

Our study team included one chief cardiovascular physician, two graduate nursing students, two nurse practitioners in charge, one associate nurse practitioner, and one chief nurse practitioner. Before the formal survey, we will conduct uniform training for surveyors so that survey members can grasp the purpose and process of the survey and ensure uniform collection methods to reduce information bias. In accordance with the findings of Biering et al., 84.5% (528/625) of patients with ischemic heart disease returned to work at 3 months after PCI [18]. Thus, this study selected to gather data in the cardiology clinic 3 months after PCI ( i.e., patient review) on patients' return to work, fatigue, return to work self-efficacy, and social support. After data collection, data were entered by two nursing postgraduates in pairs using dual computers to ensure the accuracy and completeness of the data. Professional statisticians were consulted during the data analysis phase so as to ensure the accuracy of the analysis results.

**4.6 Statistical methods**

A researcher who upheld tight confidentiality of the assigned data performed statistical analysis using the SPSS 25.0 software package. Information about counts was given as frequency and percentage. The mean and standard deviation were used to describe continuous measures that matched a normal distribution, and median (P25, P75) was used to describe those that did not. Using independent samples t-tests or Mann-Whitney U-tests, continuous variables were compared between groups of categorical variables. For comparing count information data between groups, the chi-square test, Fisher exact test, and Wilcoxon rank sum test were utilized. The multivariable logistic regression model was used to examine the independent influencing factors of returning to work. A P value of 0.05 or lower signifies statistical significance throughout the analysis.

**4.7 Technology roadmap of this study**

**
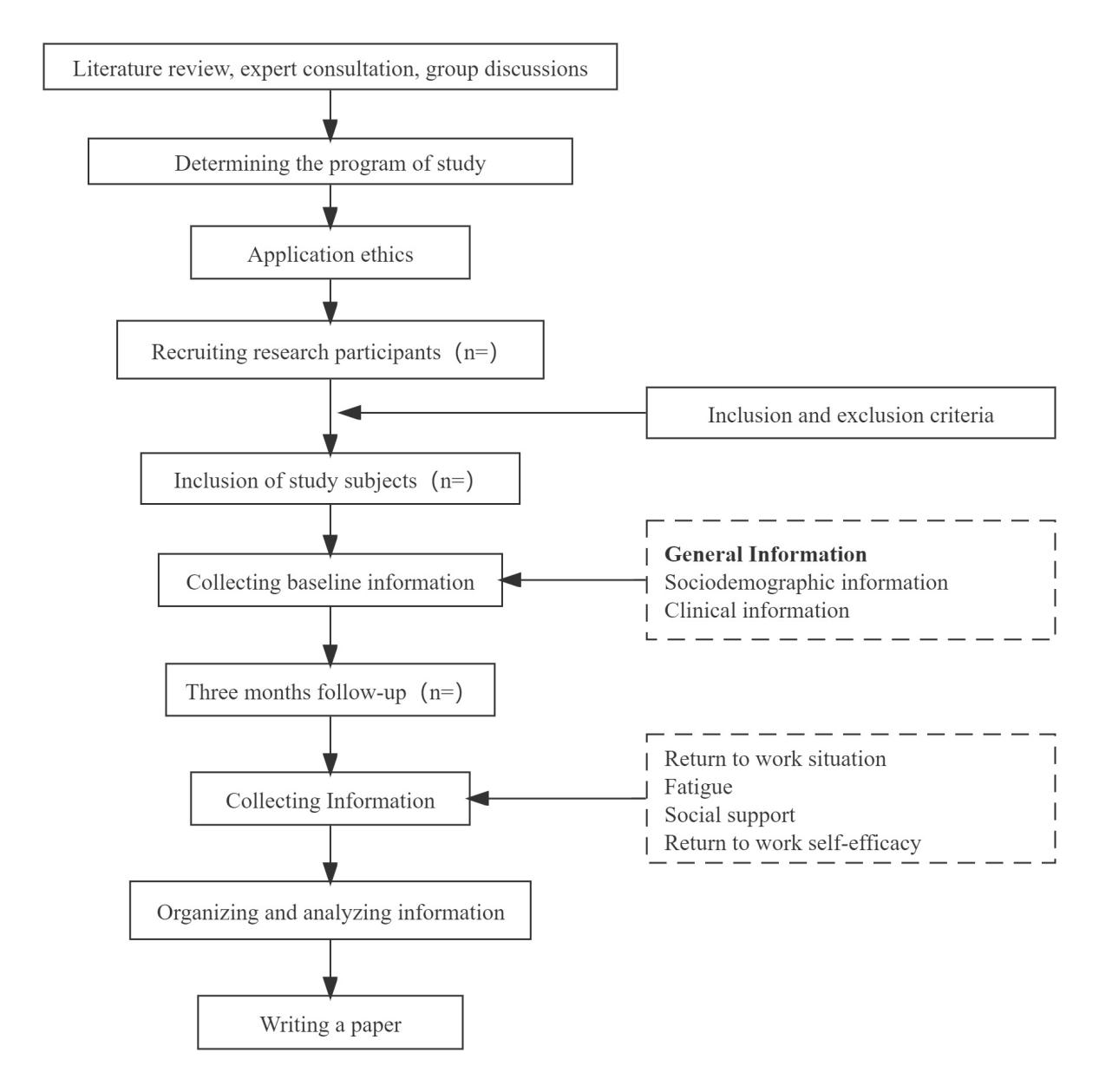
**

**5 Significance of the study**

This study can provide theoretical and practical basis for the development of return-to-work programs for young and middle-aged patients with coronary heart disease after PCI by understanding the current status of return-to-work and analyzing the factors influencing return-to-work.

**6 Expected progress and research results**

(1) Through this study, we will provide theoretical and practical basis for the development of intervention programs for the return to work of young and middle-aged patients.with CHD;

(2) Publish 1-2 papers in core or statistical source journals;

(3) Participate in one domestic academic conference for exchange.

**References**

[14] Chen B.Sample size methodology for multivariate analysis-synthetic estimate method for sample size in multivariate analysis. Injury Medicine.2012;1(4):58-60.doi:10.3868/j.issn.2095-1566.2012.04.012.

[15] Gao LP,Zhu XQ,Zhao H,Jiao HM,Chen DX.Studies on internal consistency and test-retest reliability of brief fatigue inventory in cancer patients.Nurs J Chin PLA.2009;26(8):1-3.doi:10.3969/j.issn.1008-9993.2009.08.001.

[16] Xiao SY.Theoretical basis and research application of the social support rating scale.Journal of Clinical Psychiatry.1994;(2):98-100.

[17] Gao YX,Qu QR,Wang QR,et al.Chinese Translation of the Return-To-Work Self-Efficacy Questionnaire in Cancer Patients and Its Reliability and Validity Test.Nurs J Chin PLA,2021,38(7):52-55.

[18] Biering K,Lund T,Andersen JH,et al.Effect of psychosocial work environment on sickness absence among patients treated for ischemic heart disease[J].J Occup Rehabil,2015,25(4):776-782.

**7 Research Base**

**7.1 Study and work experience of the main project leader:**

(1) 2019.09-2022.06 Master's degree, School of Nursing, Soochow University

(2) 2022.09-present Nurse, Department of Cardiology, Affiliated Hospital of Jiangnan University

**7.2 Scientific research and awards of key project team members**

(1) Relationship between dynamic changes of peri-procedure anxiety and short-term prognosis in patients undergoing elective percutaneous coronary intervention for coronary heart disease: A single-center, prospective study.PLoS ONE,2022,17(4): e0266006.

(2)Development and reliability and validity test of secondary prevention cognitive level scale for patients with coronary heart disease.CHINESE NURSING RESEARCH,2022,36(02):217-223.

(3)Awareness of secondary prevention in patients with coronary heart disease after percutaneous coronaryintervention:A qualitative study.Nursing of Integrated Traditional Chinese and Western Medicine,2020,6(07):49-53.

(4) Independent predictive effect of perioperative anxiety dynamic changes on postoperative adverse cardiovascular events in patients receiving elective PCI.Chinese Mental Health Journal,2022,36(02):111-117.

(5)Third Prize of Cardiovascular Academic Paper of Jiangsu Province, Jiangsu Nursing Society.2021.

(6)1 patent for utility model.

**8 Funding budget**

| **Item** | **Request for funding(yuan)** | **Remarks (calculation basis and description)** |
| --- | --- | --- |
| Equipment purchase cost | 100 | Signature pens |
| Energy and material costs |  |  |
| Test outsourcing fees |  |  |
| Materials, printing costs | 300 | Questionnaire and literature printing |
| Rental fees |  |  |
| Travel expenses |  |  |
| Identification and acceptance fees |  |  |
| Management fee |  |  |
| Other expenses | 3600 | Dissertation publication fees and other unforeseen contingencies |
| Total | 4000 | - |
